# Editorial: Advancements in mechanical ventilation: understanding physiology to mitigate complications

**DOI:** 10.3389/fmed.2026.1920884

**Published:** 2026-07-16

**Authors:** Luigi Vetrugno, Adrián Gallardo

**Affiliations:** 1 Department of Anesthesia and Intensive Care, Health Integrated Agency of Friuli Centrale, Tolmezzo, Italy; 2 Department of Kinesiology and Respiratory Care, “Sanatorio Clínica Modelo de Morón”, Morón, Argentina; 3 Department of Health Sciences, Kinesiology and Physiatry, National University of La Matanza, San Justo, Argentina; 4 Grupo Internacional de Ventilación Mecánica, WeVent, Viña del Mar, Chile

**Keywords:** mechanical ventilation, pediatric ventilation, personalized ventilation, positive end-expiratory pressure, respiratory mechanics, ventilator-induced diaphragmatic dysfunction, ventilator-induced lung injury

The paradigm of mechanical ventilation has undergone a substantial evolution over the past two decades—and particularly in recent years—transitioning from rigid, protocolized strategies based primarily on predicted body weight toward precision ventilation: individualized, physiology-guided, and driven by respiratory mechanics. The current goal is no longer merely to ensure adequate gas exchange, but also to mitigate two interconnected deleterious phenomena: ventilator-induced lung injury, VILI, and ventilator-induced diaphragmatic dysfunction, VIDD (1). In this Research Topic, several articles address this evolving field through the integration of pulmonary physiology, respiratory mechanics, robust methodological designs, and clinical prediction strategies aimed at personalizing respiratory support across different settings, from the operating room to the intensive care unit, ICU.

In their review article, Merola et al. synthesize the available evidence on how personalization of mechanical ventilation—grounded in real-time monitoring of the intrinsic mechanical properties of the respiratory system—may represent a cornerstone of VILI prevention. The review highlights that mechanical variables such as driving pressure, stress, and strain cannot be generalized; rather, optimization of tidal volume and positive end-expiratory pressure, PEEP, should be adapted to each patient's respiratory mechanics. The article naturally raises two questions: How can this conceptual personalization be standardized in high-turnover units without falling back into the oversimplification of older protocols? Is the lack of bedside reproducibility the true enemy of physiology-based ventilation?

Translating this philosophy into applied mathematical physiology at the bedside, Tontu et al. present a cross-sectional observational clinical study in patients with acute respiratory distress syndrome, ARDS, in which they investigated how fine-tuning ventilator settings, particularly inspiratory rise time, modifies mechanical power—a concept that is increasingly discussed but still insufficiently explored in clinical practice. Using repeated-measures analytical models, they demonstrated that shortening rise time significantly increases mechanical power in volume-controlled modes, while producing an opposite effect in pressure-controlled modes, *p* < 0.05. This finding invites us to look beyond the direct mechanical effect: although changes in rise time modify the delivery of physical energy, how do these acute flow variations affect the comfort of the awake patient, neural drive, and the risk of clinically relevant asynchronies such as double-triggering? Therefore, any reduction in mechanical power should be interpreted in parallel with its potential effects on patient comfort, neural drive, and patient–ventilator synchrony.

This approach to pulmonary and diaphragmatic protection also extends to the operating room, including vulnerable populations such as pediatric patients and infants. Two randomized controlled trials illustrate this principle. First, Yue et al. conducted a prospective study in young infants undergoing laparoscopic surgery. The authors demonstrated that a lung-protective ventilation strategy combined with alveolar recruitment maneuvers significantly reduced the incidence of intraoperative atelectasis compared with conventional ventilation, as assessed at different time points during the intraoperative period, *p* = 0.01, *p* = 0.0005, and *p* < 0.001. In a complementary surgical scenario, Wang et al. evaluated children undergoing thoracic surgery and assessed the effects of pressure-controlled ventilation with volume-guaranteed mode, PCV-VG. Using repeated-measures analysis of variance and assessment of mean differences, the study showed that PCV-VG maintained lower peak inspiratory pressure and slightly improved perioperative dynamic compliance compared with conventional volume-controlled ventilation.

The success of a personalized strategy lies not only in protecting the lung, but also in preserving the respiratory muscle pump. Andrade-Rebolledo et al. present a comprehensive systematic review and meta-analysis on the impact of inspiratory muscle training in patients undergoing prolonged weaning. Synthesizing the evidence through standardized mean differences and random-effects models, the authors found that inspiratory muscle training increases respiratory muscle strength and improves the rate of successful weaning. Although the study was not designed to directly quantify diaphragmatic dysfunction, its findings suggest that respiratory muscle training may help counteract mechanisms involved in ventilator-induced diaphragmatic dysfunction.

This process of physiological personalization culminates at the moment of definitive ventilator withdrawal. Zeng et al. contribute to this Research Topic with a methodologically rigorous systematic review on risk prediction models for extubation failure in critically ill patients. By analyzing the characteristics of existing models through evaluation of the area under the receiver operating characteristic curve, together with sensitivity and specificity values, the authors underscore the urgent need to integrate dynamic physiological variables and multivariate scores to more accurately estimate the probability of therapeutic success. They highlight that variables such as duration of mechanical ventilation, Glasgow Coma Scale score, APACHE II score, age, and hemoglobin are among the most frequently cited predictors associated with outcomes. While the findings and methodology are consistent, we cannot overlook the fact that these mathematical models—designed to capture the dynamic complexity of the interaction between central drive and muscular effort—do not always align with the characteristics of each individual patient. Statistical models can support, but not replace, individualized clinical judgment. Furthermore, upon closer examination of the included studies, only two performed both internal and external validation; three employed machine learning, while 11 used traditional modeling methods, reflecting a degree of heterogeneity that warrants careful interpretation of the findings.

The articles comprising this Research Topic collectively emphasize that mechanical ventilation is no longer a static supportive intervention, but rather a dynamic, individualized, and adaptive therapy at every stage of care. Together, these contributions provide clinicians with a useful framework for translating respiratory physiology into personalized protective practice at the bedside.

## References

[1] Vetrugno L Orso D Corradi F Zani G Spadaro S Meroi F . Diaphragm ultrasound evaluation during weaning from mechanical ventilation in COVID-19 patients: a pragmatic, cross-section, multicenter study. Respir Res. (2022) 23:210. doi: 10.1186/s12931-022-02138-y 35989352 PMC9392990

